# Measuring Staff Attitudes to Coercion in Poland

**DOI:** 10.3389/fpsyt.2021.745215

**Published:** 2021-11-16

**Authors:** Jakub Lickiewicz, Tonje Lossius Husum, Torleif Ruud, Johan Siqveland, Zofia Musiał, Marta Makara-Studzińska

**Affiliations:** ^1^Department of Health Psychology, Jagiellonian University Medical College, Kraków, Poland; ^2^Centre for Medical Ethics, The University of Oslo, Oslo, Norway; ^3^Faculty of Health Sciences, Oslo Metropolitan University, Oslo, Norway; ^4^Mental Health Service, Akershus University Hospital, Lørenskog, Norway; ^5^Institute of Clinical Medicine, The University of Oslo, Oslo, Norway; ^6^Department of Clinical Nursing, Jagiellonian University Medical College, Krakow, Poland

**Keywords:** mental health, staff, attitudes, coercion, psychometrics

## Abstract

**Introduction:** Coercion can be defined as the use of force to limit a person's choices. In Poland, coercive measures may tend to be overused. However, there is limited information regarding the attitudes of nurses toward coercion in psychiatric settings and the factors influencing any decisions to use coercion.

**Aims:** To validate the Staff Attitudes to Coercion Scale (SACS) for a group of psychiatric nurses and psychiatrists, to compare the said with the original Norwegian SACS version, and to compare nurses' attitudes with those displayed by psychiatrists. A second aim was to understand the relationship between self-efficacy and attitudes to coercion.

**Method:** We surveyed 351 psychiatric nurses and psychiatrists rating SACS and GSES (General Self Efficacy Scale). We validated the SACS factor structure using confirmatory principal component factor analysis, calculated the internal consistency of subscales, and analyzed the test-retest reliability and face validity of the subscales themselves. Further, we analyzed the differences in attitudes toward coercion between nurses and psychiatrists, as well as whether there was an association between GSES and the SACS subscales. We compared the means on the SACS items between three countries—Germany, Norway, and Poland.

**Results:** The confirmatory factor analysis of the Polish version of SACS found the same factor structure with three factors as was displayed in the original Norwegian SACS, except that one item was loaded on another factor. Internal consistency was acceptable for the factors on coercion as security and the coercion as offending, and unacceptable for the factor on coercion as treatment. Test-retest reliability was excellent for all the three subscales. Face validity was high for the factor coercion as security, partly present for coercion as offending, and not present for coercion as treatment. The subscale Coercion as Treatment was rated significantly higher by nurses than by psychiatrists, but there was no difference for the two other subscales. There was no significant association between the General Self-Efficacy Scale and any of the SACS subscales. The biggest differences in attitudes toward forms of coercion was noted between Poland and Germany.

**Discussion:** The three-factor structure of SACS was the best solution for the Polish nurses and psychiatrists. The attitudes toward coercion differed between the two groups, but a low correlation was computed for the SACS subscales and self-efficacy. There is a cultural diversity visible amongst the three countries examined. Reduction in the use of coercion is a priority worldwide. More knowledge about the process involved in using coercive measures may contribute to this. The use of coercive interventions may harm patients and threaten patients' rights. Thus, education is needed for pre-service and in-service nurses alike.

## Introduction

The use of coercion in mental health care has been debated for decades, because it challenges the fundamental medical ethics principles of respect for patient autonomy, beneficence, non-maleficence, and justice (1, 2). As a consequence, there is increasing attention and concern about the use of coercion in mental health care and the need to shift to a human-rights-based form of mental health care based on voluntariness (3). Previous research has repeatedly shown differences in the use of coercive measures in psychiatric facilities among otherwise comparable wards, hospitals, geographical areas, and countries (4, 5).

Coercion can be defined as the use of force to limit a person's choices (6), for instance, through involuntary hospitalization, compulsory medication, or the use of containment procedures such as seclusion and restraints (7). Current practices in mental health care seem to be based on “experienced-based practices” developed locally, as opposed to best-practices or evidence-based practices (8, 9).

The explanation for these differences in practice is still not fully understood, but generally thought to be involved are the differences in nursing staff attitudes toward the use of coercion in care (10, 11). Nursing staff positive attitudes toward coercive measures can also make it difficult to change the practices employed in mental health care (12).

Attitudes are involved in our interpretation of our environment and the choice as to the behaviors we display. Researchers disagree on the description of attitudes, but according to Erwin (13), the most widely used definition describes attitudes as “learned predispositions to think, feel and behave in a specific normative manner to a certain object”. The core of the definition is the three-component view. According to the three-component view, attitudes involve affective, behavioral, and cognitive aspects (13).

Another factor related to attitudes toward patient aggressive behavior and, probably indirectly, to coercive measures is self-efficacy. Self-efficacy influences personnel attitudes toward aggression. There is a correlation between knowledge in dealing with difficult behaviors and medical personnel self-efficacy (14, 15). Training on the care of aggressive and disturbed patients might influence personnel attitudes toward seclusion (16). It is possible that a lack of knowledge and low self-efficacy might lead to an overuse of coercive measures.

Research into the attitudes of mental health staff in relation to the use of coercive interventions began in the 1970s (17, 18). In the 1990s, the amount of research on mental health staff attitudes to containment measures had increased (19). In the 21st century, there has been continued interest in mental health care staff attitudes toward coercion (11, 20).

In 2008, the Staff Attitude to Coercion Scale (SACS) was developed in Norway (21). Since then, the scale has been translated into several languages and has been shown to be an acceptable instrument to research staff attitudes toward coercion in mental health care (22–24).

In Poland, the use of coercive measures is regulated by the Polish Mental Health Act (1994) (25), according to which adequate coercive measures include:

- holding, i.e., the temporary use of physical force to immobilize a patient,
- compulsory administration of drugs, both on an *ad hoc* and planned basis,
- mechanical restraints, which involve incapacitating a patient with the use of belts or other technical means,
- seclusion, which means placing the patient in solitary, in a specially adapted room (26).

Only medical doctors (including psychiatrists) may decide which type of coercive measure should be used in each situation. According to the Polish Mental Health Act, the doctor is responsible for controlling the whole procedure. However, the doctor is not necessarily involved in implementing the coercive measure, which is usually done by nurses. It might be expected, therefore, that nurses have different attitudes toward using coercive measures than do doctors.

There is limited information on the attitudes of Polish nurses toward coercion in psychiatric settings and the factors influencing any decisions to use it (27, 28). To measure staff attitudes and compare findings among countries, a validated tool was needed. An earlier study of some psychometric properties of a Polish version of SACS had been done (29). However, there is a need for a replication of these findings and analyses of the additional psychometric properties of the Polish version of SACS.

The primary aims of this study were to examine the factor structure of the Polish version of SACS, to examine the internal consistency of these factors, and to examine the reliability and face validity of SACS. The secondary aims were to compare attitudes toward coercion between Polish nurses and psychiatrists, and to examine if there was any association between self-efficacy and attitudes toward coercion.

## Methods

### Design

The study is an explorative study using several samples of completed SACS for analyses of the psychometric properties and questions listed under the aims outlined above. Psychometric properties are analyzed and interpreted according to the COSMIN standards (30). The research was approved by approved by Bioethical Commission of the Jagiellonian University Collegium Medicum (no. 122.6120.332.2016).

### Samples

The sample consisted of 351 participants (342 nurses and nine psychiatrists) from three provinces in Poland. The sample was fairly similar to the one in the original Norwegian study (21). There were 313 female and 38 male participants with a mean age of 40.4 (SD 9.2). Their mean work experience was 16.7 years (SD 10.2), and their mean work experience in psychiatry was 15.6 years (SD 10.2).

A subsample of 27 nurses also completed SACS again 2 weeks later for a reliability test-retest. Their mean age was 44.2 years (SD 7.5), and they were mostly females (92.6%). Another subsample of 113 nurses completed a questionnaire on self-efficacy. Their mean age was 43.7 years (SD 7.8), and there were 104 females (92.0%) and nine males (8.0%).

A sample of 67 psychiatric medical personnel completed the sorting of items for face validity. Their mean work experience was 23.5 years (SD 9.0), with their mean work experience in psychiatry being 19.2 years (SD 10.7).

### Measurements

The Staff Attitude to Coercion Scale (SACS) was developed and validated as a questionnaire measuring the cognitive component of mental health professionals' attitudes to coercion. It is a self-report questionnaire of 15 items, assessed by means of the 5-point Likert scale. It has three subscales: a pragmatic attitude (Coercion as Care and Security), a critical attitude (Coercion as Offending), and a positive attitude (Coercion as Treatment), with sufficient reliability demonstrated for all three subscales (Cronbach's alpha 0.69–0.73) (21). Similar results have been found in other adaptations of SACS, including in Poland (29, 31). The subscales are scored as the mean of the corresponding items (21, 23). There were no missing SACS data from any of the 351 respondents.

The General Self-Efficacy Scale (GSES) was used to assess perceived self-efficacy regarding coping and adaptation abilities in both daily activities and isolated stressful events. The tool number 10 items, scored from 1 (No) to 4 (Yes). Reliability for the GSES was previously reported as a Cronbach's alpha of 0.78 (32). In our sample, the Cronbach's alpha was calculated as 0.83.

### Data Collection

Researchers recruited medical personnel *via* an official request directed to hospital administrators in three locations, with all three administrators agreeing to allow their medical personnel to participate. The participants completed a paper-based consent form and a paper-based questionnaire during work breaks.

For the face validity data test, an online survey using the Lime Survey tool was conducted (33). The medical personnel were asked to sort each SACS item into one of three factors: coercion as security, coercion as offense, or coercion as treatment.

### Data Analyses

Descriptive data for items and subscales were reported as means (SD) (Table 1). We used confirmatory factor analyses to determine if the Polish sample contained the same factor structure of SACS as the original Norwegian sample (34, 35). The Polish sample was adequate for factor analysis as the Kaiser-Meyer-Olkin measure of sampling adequacy was 0.717 (36). Because the Norwegian sample indicated three factors for the SACS, we specified three factors in a principal component factor analysis with Varimax rotation (21, 30). Internal consistency for each factor was analyzed by calculating Cronbach's alpha. For the alpha, the team interpreted the degree of internal consistency as suggested in the guidelines by Cicchetti (1994) with the levels of unacceptable (below 0.70), fair (0.70–0.79), good (0.80–0.89), and excellent (0.90 and above) (37).

**Table 1:** Mean and standard deviation for staff attitudes to coercion scale items and subscales (*N* = 351).

**Items**	**Mean**	**SD**
1 Use of coercion is necessary as protection in dangerous situations	4.46	0.80
2 For security reasons coercion must sometimes be used	4.48	0.72
3 Use of coercion can harm the therapeutic relationship	3.15	1.11
4 Use of coercion is a declaration of failure on the part of the mental health services	1.92	0.98
5 Coercion may represent care and protection	3.83	1.01
6 More coercion should be used in treatment	2.51	1.07
7 Coercion may prevent the development of a dangerous situation	4.10	0.96
8 Coercion violates the patients' integrity	2.92	1.17
9 For severely ill patients' coercion may represent safety	4.25	0.88
10 Patients without insight require the use of coercion	2.42	1.03
11 Use of coercion is necessary toward dangerous and aggressive patients	4.35	0.87
12 Regressive patients require the use of coercion	2.38	1.25
13 Too much coercion is used in treatment	2.36	0.84
14 Scarce resources lead to more use of coercion	2.97	1.21
15 Coercion could have been noticeably reduced, giving more time and personal contact	3.18	1.21
**Subscales**		
Offending	2.75	0.69
Security	4.25	0.58
Treatment	2.44	0.73

According to the COSMIN standards, test-retest reliability was calculated as weighted kappa for items as ordinal scales, and as intra-class correlation coefficients (ICC) for the three SACS subscales (30). The estimation of the weighted kappa used linear weights. ICC was calculated by means of a one-way random effects model where the effects are random and the average measures are reported. We interpreted the weighted kappa and ICC according to Cicchetti's guidelines (1994) with levels of poor (below 0.40), fair (0.40–0.59), good (0.60–0.74), and excellent (0.75 and above) (37).

The results for the test of face validity were shown in a frequency table as the distribution of items on the three factors identified in the confirmatory factor analysis. In the COSMIN standards, face validity is defined as the degree to which the items of an instrument look as though they are an adequate reflection of the construct to be measured.

The association between GSES and each SACS subscale was analyzed using Pearson's correlation coefficients. Differences in attitudes between nurses and psychiatrists were analyzed by examining the confidence intervals (CI) and here due to the small subsample of psychiatrists. All data analyses were conducted using SPSS for Windows, version 27 (38).

## Results

### Factor Structure and Internal Consistency of Factors

The results of the confirmatory factor analysis are presented in Table 2; with the factor loadings of individual items, the percentage of variance accounted for by each factor, and the internal consistency of each factor calculated as the Cronbach alpha coefficient. The factor structure was similar to the factor structure in the original factor analysis in Norway, except that item 4 loaded highest on factor 3 instead of on factor 1. Cronbach's alpha was fair for factors 1 and 2, and unacceptable for factor 3. Pearsons' correlations between the SACS subscales were weak between Offending and Security (*R* = 0.11 *p* < 0.03) between Offending and Treatment (*R* = 0.15 *p* < 0.003), and between Treatment and Security (*R* = 0.11 *p* < 0.04). In the original study, a five-factor model was also examined with exploratory factor analysis (21). In the current study, we also carried out an exploratory factor analysis, which gave a solution with five factors explaining 60% of the variance. However, the Cronbach alpha was fair for the first two factors (0.74 and 0.70) and unacceptable for the other three (0.27–0.38). Thus, we retained the three-factor model from the confirmatory factor analysis.

**Table 2:** Confirmatory factor analysis of three factors of staff attitudes to coercion scale: Principal components analysis with varimax rotation.

**Items**	**Factors**		
	**1**	**2**	**3**
1 Use of coercion is necessary as protection in dangerous situations	**0.755**	−0.024	0.183
2 For security reasons coercion must sometimes be used	**0.735**	0.039	0.002
9 For severely ill patients' coercion may represent safety	**0.679**	0.092	−0.152
7 Coercion may prevent the development of a dangerous situation	**0.648**	−0.044	0.117
11 Use of coercion is necessary in relation to dangerous and aggressive patients	**0.631**	−0.101	0.006
5 Coercion may represent care and protection	**0.506**	−0.042	0.021
14 Scarce resources lead to more use of coercion	0.075	**0.781**	0.156
15 Coercion could have been much reduced, giving more time and personal contact	−0.119	**0.780**	0.183
8 Coercion violates the patients' integrity	−0.060	**0.677**	−0.044
3 Use of coercion can harm the therapeutic relationship	0.148	**0.584**	−0.144
13 Too much coercion is used in treatment	−0.175	**0.511**	0.103
6 More coercion should be used in treatment	0.134	−0.166	**0.703**
10 Patients without insight require the use of coercion	−0.024	0.050	**0.703**
12 Regressive patients require the use of coercion	0.105	0.205	**0.414**
4 Use of coercion is a declaration of failure on the part of the mental health services	−0.373	0.232	**0.413**
Variance explained (total 45.5%)	**19.3%**	**16.2%**	**10.0%**
Internal consistency (Cronbach's alpha)	**0.74**	**0.71**	**0.39**

*Bold values represent loadings on subscale*.

### Test-Retest Reliability

Test-retest showing the reliability of each item and the three subscales is presented in Table 3. The reliability was excellent (0.75 or above) for all the three subscales. The reliability for items was excellent (0.75 or above) for eight items, good (0.60–0.74) for six items, and fair (0.40–0.59) for one item.

**Table 3:** Reliability (test-retest) of items and subscales of staff attitudes to coercion scale (*N* = 27).

**Items**	**Weighted kappa**
1 Use of coercion is necessary as protection in dangerous situations	0.96
2 For security reasons coercion must sometimes be used	0.72
3 Use of coercion can harm the therapeutic relationship	0.66
4 Use of coercion is a declaration of failure on the part of the mental health services	0.47
5 Coercion may represent care and protection	0.60
6 More coercion should be used in treatment	0.74
7 Coercion may prevent the development of a dangerous situation	0.76
8 Coercion violates the patient's integrity	0.78
9 For severely ill patient's coercion may represent safety	0.69
10 Patients without insight require the use of coercion	0.83
11 Use of coercion is necessary in relation to dangerous and aggressive patients	0.69
12 Regressive patients require the use of coercion	0.81
13 Too much coercion is used in treatment	0.75
14 Scarce resources lead to more use of coercion	0.87
15 Coercion could have been much reduced, giving more time and personal contact	0.93
**Subscales**	**ICC**
Offending	0.97
Security	0.97
Treatment	0.96

*The estimation of the weighted kappa employs linear weights. ICC is calculated on the basis of a one-way random effects model where people effects are random and the average measurements are reported*.

### Face Validity

Table 4 shows the results of the face validity test; with items sorted on the three factors from the confirmatory factor analysis. All the six items (100%) of Coercion as Security were sorted to this factor with a high percentage for each item. Three of the five items (60%) of Coercion as Offending were sorted to this factor with lower differences in percentage, one of these with the same percentage as another factor. None of the four items (0%) of Coercion as Treatment was sorted with the highest percentage to this factor; except for the six items of Coercion as Security. The rest of the items had a much more even distribution across the three factors. As Table 4 shows, 12 of the items were sorted to with their highest percentage going to Coercion as Security, four items to Coercion as Offending, and no items to Coercion as Treatment.

**Table 4:** Face validity of staff attitude to coercion scale (SACS): Sorting of items (%) on three factors by 67 medical personnel in psychiatric departments.

**Item distribution from confirmatory factor analysis**	**Item distribution from sorting on factors**		
	**1 Security**	**2 Offending**	**3 Treatment**
**Factor 1. Coercion as security**			
1 Use of coercion is necessary as protection in dangerous situations	**91.0%**	0.0%	9.0%
2 For security reasons coercion must sometimes be used	**91.0%**	1.5%	7.5%
5 Coercion may represent care and protection	**91.0%**	1.5%	7.5%
7 Coercion may prevent the development of a dangerous situation	**94.0%**	1.5%	4.5%
9 For severely ill patients' coercion may represent safety	**83.6%**	3.0%	13.4%
11 Use of coercion is necessary in relation to dangerous and aggressive patients	**83.6%**	0.0%	16.4%
**Factor 2. Coercion as offending**			
3 Use of coercion can harm the therapeutic relationship	31.3%	**37.3%**	31.3%
8 Coercion violates the patients' integrity	44.8%	**46.3%**	9.0%
13 Too much coercion is used in treatment	**37.3%**	**37.3%**	25.4%
14 Scarce resources lead to more use of coercion	**44.8%**	34.3%	20.9%
15 Coercion could have been much reduced, giving more time and personal contact	**59.7%**	10.4%	29.9%
**Factor 3. Coercion as treatment**			
4 Use of coercion is a declaration of failure on the part of the mental health services	**41.8%**	26.9%	31.3%
6 More coercion should be used in treatment	**43.3%**	37.3%	19.4%
10 Patients without insight require the use of coercion	**47.8%**	16.4%	35.8%
12 Regressive patients require the use of coercion	29.9%	**44.8%**	25.4%

*Bold values represent loadings on subscale*.

### Differences in Attitudes Between Psychiatrists and Nurses

In examining confidence intervals (95%), there was a statistically significant difference in the Coercion as Treatment scores between nurses and psychiatrists. Nurses rated this subscale higher [2.45 (2.38–2.53)] than did physicians [1.78 (1.46–2.09)]. For the two other subscales, no statistically significant differences were found between the two groups.

### Association Between Self-Efficacy and Attitudes to Coercion

The Pearson's correlation coefficients between the GSES and the SACS subscales were 0.02 (*p* = 0.816) for Coercion as Offending, −0.04 (*p* = 0.715) for Coercion as Security, and −0.06 (*p* = 0.546) for Coercion as Treatment.

## Discussion

The confirmatory factor analysis of the Polish version of SACS found the same factor structure in the case of three factors as in the original Norwegian SACS, except that one item loaded on another factor. Internal consistency was acceptable for the factors on Coercion as Security and Coercion as Offending, and unacceptable for the factor on Coercion as Treatment. Test-retest reliability was excellent for all the three subscales. Face validity was high for the factor Coercion as Security, partly present for Coercion as Offending, and not present for Coercion as Treatment. The subscale Coercion as Treatment was rated significantly higher by nurses than by psychiatrists, but there was no difference for the two other subscales. There was no significant association between the General Self-Efficacy Scale and any of the SACS subscales.

### Factor Structure and Internal Consistency of Factors

In this sample, the factor structure for Polish medical personnel was similar to the original version of the SACS, with one exception—item 4 (21). When comparing the three-vs.-five-factors model, the three-factor models were chosen by Husum et al. (21). However, in the German validation, one-factor models were perceived as being more accurate (23). In the current study, we decided to keep a three-factor model. The three-factor structure for the Polish group was similar to the original construction of the SACS (21), and named the same as in the original validation: Coercion as Offending, Coercion as Security, and Coercion as Treatment. Five factors seem to be less useful in practice and more complicated to comprehend, and thus, were not used.

In the first study on SACS in Poland, the same structure was found in factors related to so-called “pragmatic” attitudes (29). For many years, coercion was perceived as an integral part of Polish psychiatry. However, the original SACS study was conducted in 2008. There is a possibility of changes in attitudes toward coercion in the perception of Norwegian medical personnel. There is still a lack of knowledge in understanding how time and cultural changes might affect the perception of coercion. Attitudes may be changing. Attitudes may have changed proportionally as opposition to the use of coercion has increased.

In a previous study by Kiejna et al. (29), the three-factor solution was identical to the Norwegian factors (29). In the current study, the confirmatory factor analysis with nurses and psychiatrists as respondents resulted in the same factors as the Norwegian sample, except that item 4 loaded highest on Treatment instead of Offending. The factor structure in the Polish sample is the same except for item 4 (“Use of coercion is a declaration of failure on the part of the mental health services”). There might possibly be a few explanations for it.

The first explanation might be connected with a real difference in attitudes between the countries. A second reason might be related to the translation process and the understanding of some of the item terms. For example, some of the meaning of the words might differ between Polish and the original Norwegian version of SACS, and, this might possibly be confusing for the respondents. However, only for the Polish translation was this item problematic—the SACS factor structure was similar to Norwegian in a study conducted in Iran (31). Cross-cultural aspects of translation might be an interesting issue for future research (39). More research is needed on the issue, including the possibility of removing the item from SACS. It might also be interesting to compare attitudes toward coercion in different countries, taking into account economical, historical, and political factors.

Our research suggests the need to delete item 4 from the Polish version of SACS because of its ambiguity and influence on other items. For this reason, validation of the translation process and cultural sensitivity awareness seem to be important factors in adapting the SACS.

### Test-Retest Reliability

Test-retest showed good SACS stability. Again, item 4 obtained a relatively low test-retest stability (0.56), which is reason enough to remove it from the final version of the Polish SACS. Another issue is the difference between the dimensions from the one obtained in other studies. The three factors obtained in the original research (21) and, consequently, the more complex perception of coercion may be related to differences in the health care curriculum. One of the elements is the lack of courses in the prevention of violence and aggression. Another is the education system itself, as psychiatric nursing is a specialization in Poland, which can only be undertaken after completing undergraduate studies and an appropriate period of work in the profession. Another reason is the specifics of work in different countries and the methods of treatment used, such as the medical treatment at the expense of therapy and individual patient contact. Identifying nurses' attitudes toward coercion is important to determine appropriate nursing curricular content.

### Face Validity

The attempt to confirm the construct of the extracted factors by face validity was not entirely successful. Twelve of the 15 items were sorted with their highest percentage to the factor Coercion as Security (including six items from the two other factors), and none of the items of the Coercion as Treatment factor being sorted with the highest percentage to this factor. The results indicate that the participants mostly considered coercion as security and partly as offending, and only to a very small extent as treatment. This seems to support other parts of the discussion regarding both the cultural differences and differences in time as to the validity of the results for the Norwegian SACS in 2008. However, when the nurses and psychiatrists in the current study rated their attitudes to coercion by completing SACS, the confirmatory factor analysis still found the same factors as in Norway, including the factor for Coercion as Treatment.

### Differences in Attitudes Between Psychiatrists and Nurses

There are strong arguments to select nurses as a homogenous group—in Poland, it is nurses who implement treatments, more often than do medical doctors or psychologists. For that reason, nurses' attitudes toward coercion might differ from other medical care specialists. Nurses are exposed to more violence than psychiatrists, often in situations where they apply coercive measures, which may be a reason for differences in the perception of coercion between psychiatric nurses and psychiatrists. The difference in the sample sizes reflects the difference in the number of nurses and psychiatrists in psychiatric departments; psychiatrists' attitudes are equally more important as it is they who take the decisions to employ coercion measures. Psychiatric nurses agreed more strongly than psychiatrists with the statement that more coercion should be used in treatment. Psychiatric nurses are personally involved in applying coercive measures, and thus, they become targets for aggressive behaviors more often than psychiatrists. Interestingly, there are some cultural differences in this issue. In the Norwegian study, psychiatrists showed a more authoritarian approach than did nurses (40). This may be related to the differences in the relationship between medical personnel and patients in Poland and Norway.

Currently, there is no curricular content for teaching Polish nursing students how to effectively manage patient aggression and violence (41). Due to the lack of other solutions, they may overestimate the necessity of coercive measures. There is a need for nurses' training in aggression prevention and management, based on understanding the motivation and negative consequences of coercive measures. Behavioral changes in nurses' view of aggression will lead to fewer conflicts between staff and patients, which, in turn, will lead to fewer patients being restrained (42). Thus, far, there have been limited initiatives in Poland to address aggression and violence in mental hospitals (43). It seems to be important to give nurses knowledge in areas of conflict de-escalation or through the use of alternative interventions, like e.g., Safewards, which is effective intervention to reduce violence. It also influences medical personnel attitudes toward coercion (10, 44). SACS can measure not only initial attitudes but also attitudinal changes. It is important to use evidence-based training with evaluation, which SACS can provide.

Attitudes toward coercion are conditioned by many factors, including experiences derived from working with specific types of patients. Such differences should be looked at in a global context, taking into account the specific nature of treatment in individual countries. The means in the three groups appear to be very similar; however, some differences in factor structure do emerge. This might indicate that cultural differences exist, not in understanding coercive measures *per se*, but in attitudes toward using them.

### Reflections Regarding Attitudes Toward Coercion in Different Countries

In order to reflect on any possible differences between countries in attitudes to coercion measured using SACS, we have compared published results from studies conducted in Norway, Germany, and Poland. Polish medical personnel see coercion as a part of the treatment and an acceptable solution in dealing with aggressive behaviors. The biggest differences might be noted between Poland and Germany. Geographically, the countries share a border, but they are very far apart in their perception of coercion.

The first challenge in Poland is that there are insufficient medical personnel on any one shift, which is why they might tend to overuse coercion in difficult situations (45). They are also not in possession of alternative solutions, such as Safewards. According to nurses, they are overloaded with work and cannot spend extra time with agitated patients (46), which may lead to a higher level of moral distress. This concept is related to the external constraints connected to obstacles outside of the individual, whether institutional, systemic, or situational.

The second challenge are the internal constraints located within the individuals themselves, which are described as personal limitations, failings, or weakness of will (47). Any perceived lack of self-efficacy and knowledge in dealing with difficult behaviors, in connection with work overload and institutional obstacles related to coercion, might also provoke discomfort. It might result in a vicious cycle, in which using coercive measures leads to moral distress.

To compare the attitudes toward coercive measures in different countries, the means for individual items were compared. Some differences in the means between countries were found. Norwegian medical personnel agreed that coercion might represent care and protection more than in other countries (item 5: 4.21 vs. 3.82). According to Polish medical personnel, coercion should be used more often (2.51). German medical personnel have a different point of view (item 6: 1.81). In Poland, coercion is way to deal with a dangerous situation on the ward (item 7: 4.10 for Poland, 3.52 for Germany). It can also be a solution for severely ill patients (item 9: 4.35 in Poland, 3.64 in Germany). There were differences in opinion about the violation of integrity (item 8), with the biggest difference between Poland (2.92) and Germany (3.68). A similar situation exists with items 14 and 15: (2.97 vs. 3.58) and (3.18 vs. 4.04) (21.23)

Item 4, which was problematic in the factor analysis, showed cultural differences. In item 4, German medical personnel admitted that the use of coercion might be perceived as failure (2.28), while in Poland this issue was perceived differently (1.92) (21.23).

In Norway, where the population has a normative attitude toward respect for autonomy and human rights, which may be reflected in the professionals' attitudes toward respect for patients' autonomy and human rights. A medical culture created by former authoritarian countries, like Poland, might have led to strong paternalism (27, 28). Mental health care was more paternalistic in history, but in contemporary mental health care, more collaborative work methods have been developed. A patient's rights are an important issue in modern psychiatry.

This allowed us to come to the conclusion that there is cultural diversity among the three countries compared. The reasons might be because of economic (insufficient medical personnel), and historical and education factors (no alternative de-escalation programmes). Nurses' attitudes toward coercion might prevent any excessive use of mechanical restraints (20). Observing changes in nurses' attitudes toward coercion and its overuse might alert supervisors to unwanted behaviors, such as cynicism or a low sense of personal accomplishment about patients, which are indicative of burnout (48).

### Association Between Self-Efficacy and Attitudes to Coercion

We did not find a significant association between self-efficacy and attitudes toward coercion. Undoubtedly more research is needed into this issue. Still little is known about the factors influencing attitudes and leading to decisions to use coercive measures. However, the previous research shows the relationships between training in using restraint practices though this was not something revealed by our investigations (16, 49).

General attitudes toward different situations correlate well with general behavioral patterns, but not with specific behaviors. Predicting specific actions, like the use of coercion, requires a measure of attitude toward the behavior itself, as in the reasoned action approach, which takes specific behavior as the starting point and identifies intentions, attitudes, norms, and perceived behavioral control as important determinants. Thus, to improve the mental health treatment system, it is first crucial to understand the attitudes of medical personnel toward coercion in different settings. The next step in the research should be to determine how personality factors, but also teaching curriculum and public opinion, can influence attitudes toward coercion (50).

### Strengths and Difficulties

In our study, we employed COSMIN standards for a higher level of methodological correctness (30). The study, however, has some limitations. One of them is the relatively small number of psychiatrists in the study. Another limitation might be that the test-retest was only conducted with the group of nurses, and that it may not be representative for psychiatrists.

Although we used a large sample of psychiatric nurses (*n* = 351), they were only from three districts of Poland. In this context, working ethics and hospital regulations could potentially influence the nurses' attitudes. Thus, the perspectives of nurses from other hospitals should also be considered.

Lack of knowledge, work overload, and responsibility for applying coercion might lead nurses to overuse it. Sometimes coercive measures might be the easiest, but not the best, solution.

## Conclusion

Our study adds new knowledge about staff attitudes toward coercion in mental health care. It also gives a more insightful view as to the validity of the Staff Attitude to Coercion Scale (SACS).

In our study, we suggest a three-factor model as the most effective in analyzing attitudes toward coercion in a group of psychiatric nurses and psychiatrists. According to the data obtained, the Polish version of SACS should not include item 4, because of its ambiguity. Our research did not confirm any correlation between SACS and self-efficacy, but showed differences in the attitudes of psychiatric nurses and psychiatrists.

There are some practical implications of our study. SACS appears an interesting and valuable tool that can be used within research into coercion. However, there is a need to compare attitudes in other countries and cultures. It would also be valuable to compare attitudes of other groups of nurses who use coercive measures, such as geriatric nurses. To summarize, the use of the SACS might be beneficial; however, one must be cognizant of cultural differences, not a measurement problem—as it is in the case of psychiatrists and psychiatric nurses.

## Data Availability Statement

The original contributions presented in the study are included in the article/supplementary material, further inquiries can be directed to the corresponding author.

## Ethics Statement

The studies involving human participants were reviewed and approved by Bioethical Commission of the Jagiellonian University Collegium Medicum (no. 122.6120.332.2016).

## Author Contributions

All the authors have been involved in the process of assessing studies for inclusion, analyzing results, and writing the article, except ZM, who was responsible for data collection, and MM-S, who revised the manuscript.

## Funding

This research was a part of the project Adaptation of tools used by medical personnel for measure of aggressive behaviors funded by Ministry of Science of Republic of Poland (no. 43/DBS/000066).

## Conflict of Interest

The authors declare that the research was conducted in the absence of any commercial or financial relationships that could be construed as a potential conflict of interest.

## Publisher's Note

All claims expressed in this article are solely those of the authors and do not necessarily represent those of their affiliated organizations, or those of the publisher, the editors and the reviewers. Any product that may be evaluated in this article, or claim that may be made by its manufacturer, is not guaranteed or endorsed by the publisher.

## References

[1] Beauchamp T Childress J. Principles of Biomedical Ethics. 8th ed. Oxford: Oxford University Press (2019).

[2] Luciano M Sampogna G Del Vecchio V Pingani L Palumbo C De Rosa C. Use of coercive measures in mental health practice and its impact on outcome: a critical review. Expert Rev Neurother. (2014) 14:131–41. 10.1586/14737175.2014.874286 24382132

[3] Zinkler M von Peter S. End coercion in mental health services—toward a system based on support only. Laws. (2019) 8:19. 10.3390/laws803001933351411

[4] Donisi V Tedeschi F Wahlbeck K Haaramo P Amaddeo F. Pre-discharge factors predicting readmissions of psychiatric patients: a systematic review of the literature. BMC Psychiatry. (2016) 16:449. 10.1186/s12888-016-1114-0 27986079 PMC5162092

[5] Keown P McBride O Twigg L Crepaz-Keay D Cyhlarova E Parsons H. Rates of voluntary and compulsory psychiatric in-patient treatment in England: an ecological study investigating associations with deprivation and demographics. Br J Psychiatry. (2016) 209:157–61. 10.1192/bjp.bp.115.171009 27284079

[6] Wynn DR. Coercion in psychiatric care: clinical, legal, and ethical controversies. Int J Psychiatry Clin Pract. (2006) 4:247–51. 10.1080/13651500600650026 24941142

[7] Bowers L. On conflict, containment and the relationship between them. Nurs Inq. (2006) 13:172–80. 10.1111/j.1440-1800.2006.00319.x 16918784

[8] Bowers L Alexander J Simpson A Ryan C Carr-Walker P. Cultures of psychiatry and the professional socialization process: the case of containment methods for disturbed patients. Nurse Educ Today. (2004) 24:435–42. 10.1016/j.nedt.2004.04.008 15312952

[9] Husum TL. Staff Attitudes and Use of Coercion in Acute Psychiatric Wards In Norway Dissertation Tonje Lossius Husum (2011). Available online at: https://www.tvangsforskning.no/filarkiv/File/Avhandling_Tonje_Lossius_Husum.pdf (accessed July 20, 2020)

[10] Bowers L Van Der Merwe M Nijman H Hamilton B Noorthorn E Stewart D. The practice of seclusion and time-out on english acute psychiatric wards: the city-128 study. Arch Psychiatr Nurs. (2010) 24:275–86. 10.1016/j.apnu.2009.09.003 20650373

[11] Laukkanen E Vehviläinen-Julkunen K Louheranta O Kuosmanen L. Psychiatric nursing staffs' attitudes towards the use of containment methods in psychiatric inpatient care: an integrative review. Int J Ment Health Nurs. (2019) 28:390–406. 10.1111/inm.12574 30761718

[12] Sailas E Wahlbeck K. Restraint and seclusion in psychiatric inpatient wards. Curr Opin Psychiatry. (2005) 18:555–9. 10.1097/01.yco.0000179497.46182.6f 16639118

[13] Erwin P. Attitudes and Persuasion. Attitudes Persuas. Hove: Psychology Press (2014).

[14] Ziekenhuis Ghent U Beeckman D van Meijel B. Mental Health Nurses' Attitudes and Perceived Self-Efficacy Toward Inpatient Aggression: A Cross-Sectional Study of Associations With Nurse-Related Characteristics. Perspect Psychiatr Care. (2016) 52:12–24. 10.1111/ppc.12097 25495430

[15] Hostetler T. The comparative change of self-efficacy in nursing students after verbal de-escalation education. Int J Stud Nurs. (2020) 5:103. 10.20849/ijsn.v5i1.730

[16] Kontio R Lahti M Pitkänen A Joffe G Putkonen H Hätönen H Välimäki M. (2011). Impact of eLearning course on nurses' professional competence in seclusion and restraint practices: a randomized controlled study (ISRCTN32869544). J Psychiatr Ment Health Nurs. (2011) 18:813–21. 10.1111/j.1365-2850.2011.01729.x 21985684

[17] Heyman E. Seclusion. J Psychosoc Nurs Ment Health Serv. (1987) 25:9–12. 10.3928/0279-3695-19871101-04 3694565

[18] Plutchik R Karasu TB Conte HR Siegel B Jerrett I. Toward a rationale for the seclusion process. J Nerv Ment Dis. (1978) 166:571–9. 10.1097/00005053-197808000-00004 681917

[19] Muir-Cochrane E. An investigation into nurses' perceptions of secluding patients on closed psychiatric wards. J Adv Nurs. (1996) 23:555–63. 10.1111/j.1365-2648.1996.tb00019.x 8655832

[20] Happell B Harrow A. Nurses' attitudes to the use of seclusion: a review of the literature. Int J Ment Health Nurs. (2010) 19:162–8. 10.1111/j.1447-0349.2010.00669.x 20550639

[21] Husum TL Finset A Ruud T. The Staff Attitude to Coercion Scale (SACS): reliability, validity and feasibility. Int J Law Psychiatry. (2008) 31:417–22. 10.1016/j.ijlp.2008.08.002 18817973

[22] Al-Maraira OA Hayajneh FA. Correlates of psychiatric staff's attitude toward coercion and their sociodemographic characteristics. Nurs Forum. (2020) 55:603–10. 10.1111/nuf.12476 32497275

[23] Efkemann SA Scholten M Bottlender R Juckel G Gather J. A German version of the staff attitude to coercion scale. Development and Empirical Validation. Front Psychiatry. (2021) 11:573240. 10.3389/fpsyt.2020.573240 33536947 PMC7847975

[24] Wu H-C Tang I-C Lin W-I Chang L-H. Professional values and attitude of psychiatric social workers toward involuntary hospitalization of psychiatric patients. J Soc Work. (2013) 13:419–34. 10.1177/1468017311435201

[25] Mental Health Act [Ustawa o ochronie zdrowia psychicznego] [in Polish]. Dz. U. (1994) 111:poz. 535.

[26] Karcz E Zimmermann A. Direct coercion–challenges for nursing practice. Pielegniarstwo XXI wieku. (2017) 16:58–63. 10.1515/pielxxiw-2017-0020

[27] Dlouhy M. Mental health policy in Eastern Europe: a comparative analysis of seven mental health systems. BMC Health Serv Res. (2014) 14:42. 10.1186/1472-6963-14-42 24467832 PMC3908346

[28] Winkler P Krupchanka D Roberts T Kondratova L Machu V Höschl C. A blind spot on the global mental health map: a scoping review of 25 years' development of mental health care for people with severe mental illnesses in central and eastern Europe. Lancet Psychiatry. (2017) 4:634–42. 10.1016/S2215-0366(17)30135-9 28495549

[29] Kiejna A Jakubczyk MH Gondek TM Rajba B. The Staff Attitude to Coercion Scale (SACS)-Polish adaptation. Psychiatria Polska. (2020) 54:113–24. 10.12740/PP/102438 32447360

[30] Prinsen CAC Mokkink LB Bouter LM Alonso J Patrick DL de Vet HCW. COSMIN guideline for systematic reviews of patient-reported outcome measures. Qual Life Res. (2018) 27:1147–57. 10.1007/s11136-018-1798-3 29435801 PMC5891568

[31] Arab M Gray S Hamouzadeh P Habibi F. Validation of the Staff attitude toward coercion use in treatment of mentally ill patients questionnaire in selected public psychiatric hospitals of Tehran in (2015). J Hosp. (2017) 16:31–42.

[32] Juczyński Z. Narzedzia pomiaru w promocji i psychologii zdrowia [in Polish] (Tools in Health Psychology. The Manual). Warszawa: Pracownia Testów Psychologicznych PTP (2001)

[33] Limesurvey. LimeSurvey Manual (2019). Available online at: https://manual.limesurvey.org/LimeSurvey_Manual (accessed July 20, 2020)

[34] Netemeyer RG Bearden WO Sharma S. Scaling Procedures: Issues and Applications. Thousand Oaks, CA: SAGE Publications (2003).

[35] DeVellis RF Thorpe CT. Scale Development: Theory and Applications. https://www.google.com/search?sxsrf=AOaemvJSeJjZ6E27ZMSRcovk_hQmykn2ow:1633949735549&q=Thousand+Oaks&stick=H4sIAAAAAAAAAOPgE-LUz9U3sEw2MC9R4gAxiyySLbS0spOt9POL0hPzMqsSSzLz81A4VhmpiSmFpYlFJalFxYtYeUMy8kuLE_NSFPwTs4t3sDICAGo7m7JWAAAA&sa=X&ved=2ahUKEwj0ocWMmcLzAhV44jgGHagSDZ0QmxMoAXoECFMQAw Thousand Oaks, CA: SAGE publications (2021).

[36] Pett MA Lackey NR Sullivan JJ. Making Sense of Factor Analysis: The Use of Factor Analysis for Instrument Development in Health Care Research. Thousand Oaks, CA: SAGE (2003).

[37] Cicchetti DV. Guidelines, criteria, and rules of thumb for evaluating normed and standardized assessment instruments in psychology. Psychol Assess. (1994) 6:284–90. 10.1037/1040-3590.6.4.284

[38] IBM. IBM SPSS Statistics for Windows, Version 27.0. https://www.google.com/search?sxsrf=AOaemvJQv_VAOpC-CdcTGrbWkqkoWLfe4w:1633949877918&q=Armonk&stick=H4sIAAAAAAAAAOPgE-LQz9U3MC5OtlACsyqTqlK0tLKTrfTzi9IT8zKrEksy8_NQOFYZqYkphaWJRSWpRcWLWNkci3Lz87J3sDICAPlycQ9OAAAA&sa=X&ved=2ahUKEwjX47bQmcLzAhWywjgGHXy9AOEQmxMoAXoECFsQAw Armonk, NY: IBM Corp. (2020).

[39] Goyal AK Bakshi J Panda NK Kapoor R Dharam Vir Kumar K. A hybrid method for the cross-cultural adaptation of self-report measures. Int J Appl Posit Psychol. (2021) 6:45–54. 10.1007/s41042-020-00039-3

[40] Aasland OG Husum TL Førde R Pedersen R. Between authoritarian and dialogical approaches: attitudes and opinions on coercion among professionals in mental health and addiction care in Norway. Int J Law Psychiatry. (2018) 57:106–12. 10.1016/j.ijlp.2018.02.005 29548497

[41] Lickiewicz JP. Doświadczanie agresji w pracy pielegniarskiej [in Polish] Aggression in nursing experience. Sztuka Leczenia. (2014) 29:11–22.

[42] Tomagová M Bóriková I Lepiešová M Cáp J. Nurses experience and attitudes towards inpatient aggression on psychiatric wards. Cent Eur J Nurs Midw. (2016) 7:462–9. 10.15452/CEJNM.2016.07.0016

[43] Lickiewicz J Nag T Ravnanger C Jagielski P Makara-Studzińska M. The perception of aggression towards the medical personnel of psychiatric wards in Poland and in Norway – a comparative analysis. Arch Psychiatry Psychother. (2019) 21:61–70. 10.12740/APP/110698

[44] Higgins N Meehan T Dart N Kilshaw M Fawcett L. Implementation of the safewards model in public mental health facilities: a qualitative evaluation of staff perceptions. Int J Nurs Stud. (2018) 88:114–20. 10.1016/j.ijnurstu.2018.08.008 30236863

[45] Remberk B Kosińska B Bragoszewska J Bogucka-Bonikowska A Twardowska M. Problems and strengths in psychiatric nursing in poland: current legal regulations and results of survey. Issues Ment Health Nurs. (2020) 41:339–347. 10.1080/01612840.2019.1663456 31855508

[46] Sygit-Kowalkowska E. Zmeczenie przewlekłe pielegniarek w obliczu wyzwań współczesnej opieki psychiatrycznej. Czy temperament i środowisko pracy maja znaczenie? [In polish] Chronic fatigue of nurses in view of the challenges of contemporary psychiatric care. Do temperament and work environment matter? Psychiatr Pol. (2020) 176:1–18. 10.12740/PP/OnlineFirst/118480 34997742

[47] Deschenes S Kunyk D. Situating moral distress within relational ethics. Nurs Ethics. (2020) 27:767–77. 10.1177/0969733019884621 31802709

[48] de Looff P Nijman H Didden R Embregts P. Burnout symptoms in forensic psychiatric nurses and their associations with personality, emotional intelligence and client aggression: a cross-sectional study. J Psychiatr Ment Health Nurs. (2018) 25:506–16. 10.1111/jpm.12496 30199590

[49] Krieger E Moritz S Lincoln TM Fischer R Nagel M. Coercion in psychiatry: a cross-sectional study on staff views and emotions. J Psychiatr Ment Health Nurs. (2021) 28:149–62. 10.1111/jpm.12643 32348607

[50] Ajzen I Schmidt P. Changing behavior using the theory of planned behavior. In: Hagger MS Cameron LD Hamilton K Hankonen N Lintunen T, editors. The Handbook of Behavior Change. Oxford: Oxford University Press (2020), p. 17–31.

